# Influence of Preaging Temperature on the Indentation Strength of 3Y-TZP Aged in Ambient Atmosphere

**DOI:** 10.3390/ma14112767

**Published:** 2021-05-23

**Authors:** Ki-Won Jeong, Jung-Suk Han, Gi-Uk Yang, Dae-Joon Kim

**Affiliations:** 1 Department of Prosthodontics, School of Dentistry and Dental Research Institute, Seoul National University, Seoul 03080, Korea; jkw857@gmail.com (K.-W.J.); proshan@snu.ac.kr (J.-S.H.); 2 VASIC Research Center, Department of Dentistry, School of Dentistry and Dental Research Institute, Seoul National University, Seoul 03080, Korea; clemp1234@gmail.com

**Keywords:** zirconia, aging, Vickers indentation, phase transformation, biaxial strength

## Abstract

Yttria-stabilized zirconia (3Y-TZP) containing 0.25% Al_2_O_3_, which is resistant to low temperature degradation (LTD), was aged for 10 h at 130–220 °C in air. The aged specimens were subsequently indented at loads ranging from 9.8 to 490 N using a Vickers indenter. The influence of preaging temperature on the biaxial strength of the specimens was investigated to elucidate the relationship between the extent of LTD and the strength of zirconia restorations that underwent LTD. The indented strength of the specimens increased as the preaging temperature was increased higher than 160 °C, which was accompanied by extensive t-ZrO_2_ (t) to m-ZrO_2_ (m) and c-ZrO_2_ (c) to r-ZrO_2_ (r) phase transformations. The influence of preaging temperature on the indented strength was rationalized by the residual stresses raised by the t→m transformation and the reversal of tensile residual stress on the aged specimen surface due to the indentation. The results suggested that the longevity of restorations would not be deteriorated if the aged restorations retain compressive residual stress on the surface, which corresponds to the extent of t→m phase transformation less than 52% in ambient environment.

## 1. Introduction

Zirconia is currently one of the most commonly used ceramic materials in restorative dentistry. The application of the first generation of 3 mol.% Y_2_O_3_-stabilized tetragonal zirconia polycrystal (3Y-TZP) was limited to frameworks for all-ceramic restorations, on which feldspathic porcelain was layered, owing to its insufficient translucency [1]. However, veneering is a technique sensitive and time-consuming procedure, accompanied by significant tooth reduction and a high risk of chipping, which is a major cause of failure in zirconia veneering ceramics [2,3]. To alleviate such drawbacks, the monolithic zirconia restorations are adapted [4]. However, there are concerns regarding low temperature degradation (LTD) because monolithic zirconia restorations are directly exposed to the humid oral environment and masticatory force. The extent of LTD is determined by the fraction of monoclinic phase of ZrO_2_ (m-ZrO_2_) transformed from tetragonal crystal structure of ZrO_2_ (t-ZrO_2_) during aging. Indeed, in vivo aging of 3Y-TZP in the oral environment led to ~12% of m-ZrO_2_ formation after exposing for 24 months [5]. Even 60 days of intra-oral aging of flat 3Y-TZP specimens, exposed to the oral cavity, resulted in 4.7–7.7% of m-ZrO_2_ and deteriorated the strength by 32–39.4% [6]. LTD is a phenomenon in which Y_2_O_3_-containing t-ZrO_2_ transforms to m-ZrO_2_ to deteriorate mechanical properties during aging at temperatures of 100–300 °C and is accelerated in the presence of moisture, such as in the oral environment [7].

LTD is originated from the fact that at ambient temperature t-ZrO_2_ is internally strained by the adaptation of Zr cation in a distorted eightfold coordination environment because the size of Zr^4+^ is too small to be coordinated to eight oxygen ions in the fluorite structure [7]. In the system ZrO_2_-Y_2_O_3_, a metastability of t-ZrO_2_ is achieved by the substitution of Y^3+^, whose ionic radius is larger than that of Zr^4+^, for Zr^4+^. The substitution leads to the formation of oxygen vacancies and ZrO_7_ oxygen polyhedron due to the lower valency of Y^3+^ than Zr^4+^. LTD relieves the internal stress by leaving the Zr cation in sevenfold coordination with the oxygen ions, which is found in m-ZrO_2_. The activation energy for LTD was determined to be ~83 kJ/mol [7] that is in excellent agreement with the measured and theoretical values for the ionic conductivity of 3Y-TZP whose governing mechanism is the oxygen vacancy diffusion [8,9,10]. Based on the identical activation enthalpies, it was proposed that LTD is attributed to oxygen vacancy diffusion [7,8]. That is, LTD on the surface proceeds to relieve the residual stress, which accumulates as a result of the reduction in the oxygen vacancy concentration with prolonged aging at low temperatures. The observation of LTD selectively on the anode-sided surface under an applied electric field further supports that the depletion of the positively charged oxygen vacancies leads to LTD [8,11].

A mechanism, proposed alternatively, is that O^2^^−^ originating from the dissociation of water, and not OH^−^, fills the oxygen vacancies within the crystal lattice, destabilizing the t-ZrO_2_ to lead LTD [12]. Chevalier et al. have determined the activation energy for the aging of 3Y-TZP to be 106 kJ/mol and fitted the aging kinetics in the Mehl–Avrami–Johnson law to allow a prediction of the m-ZrO_2_ fraction at the surface of the aged 3Y-TZP for a given time and temperature [13]. This led to the proposal of extrapolation of low temperature degradation rate from accelerated aging tests for the lifetime prediction of medical grade zirconia, that is, a steam sterilization at 134 °C for 5 h simulates 15–20 years at 37 °C [14]. The generally accepted extrapolation has been questioned by the fact that the extrapolation underestimates LTD in vivo [5,15] and even can lead to unacceptable conclusions about the lifetime of the zirconia-based components [16]. This is because the extrapolation depends heavily on the activation energy and the uncertainty associated with the determination of the activation energy is generally high [15,16].

The severity of LTD has been characterized by measuring the extent of t-ZrO_2_ to m-ZrO_2_ (t→m) phase transformation mainly using the change in the peak intensities of the X-ray diffraction (XRD) patterns of each phase after aging under various conditions [17,18]. Garvie and Nicholson [17] estimated the fraction of transformation by the peak intensity ratio of [m($\bar{1}$11) + m(111)]/[m($\bar{1}$11) + m(111) + t(101)], X_m_, assuming that the intensity of tetragonal t(101) (or cubic (111)) peak is equal to the sum of the intensities of monoclinic m($\bar{1}$11) and m(111) peaks. However, the linear concentration relationship is not strictly correct so that Toraya et al. [18] refined the ratio to 1.311 X_m_/(1 + 0.311 X_m_) utilizing the Garvie and Nicholson’s ratio. The nonlinear calibration was suggested because the theoretical X-ray intensities of the peaks predict an inequality of the intensities, that is, t(101) > m($\bar{1}$11) + m(111) [19]. In realty the transformation in LTD always companies with an asymmetric broadening of the t(101) peak that has been described as the formation of rhombohedral ZrO_2_ (r-ZrO_2_) [7,20] and cubic ZrO_2_ (c-ZrO_2_) [21]. The additional phases should cause an error in determining the precise extent of LTD and consequently result in the uncertainty in the determination of the activation energy involved in LTD. The error stems from the fact that the quantitative analyses are based on the simple mixtures of constitute monoclinic and tetragonal (cubic) ZrO_2_ powders [17,18,19] so that they cannot account for the formation of additional residual stress-assisted r-ZrO_2_ [7,20] and the decrease of c-ZrO_2_ [20] as LTD proceeds. It is noteworthy that the activation energy of 106 kJ/mol was obtained from the m-ZrO_2_ fraction determined by ‘a modified Garvie and Nicholson equation’ [13] and the 83 kJ/mol was calculated after stripping off the contributions of r-ZrO_2_ and c-ZrO_2_ from the intensity of t(101) peak of t-ZrO_2_ [7].

The effect of LTD on the mechanical properties has been quite controversial. Some reports state that the increase in flexural strength with LTD results from transformation toughening to induce compressive stress on the surface [22,23,24]. Conversely, the decrease in strength may be because the number of micro-cracks around the grains formed by aging is sufficient to cause an imbalance with the compressive stress [25,26,27]. Finally, the mechanical property is not affected by aging because the aging conditions, such as aging temperature or time, are not sufficient to influence the strength [28,29,30]. Thus, the influence of LTD on the flexural strength of 3Y-TZP has not been clearly elucidated.

Ceramics become fractured when the applied stress intensity exceeds the critical stress intensity factor, that is, the fracture toughness. Intrinsic flaws that control the strength can be introduced during the fabrication process such as powder compaction, forming, drying, firing, and cooling [31]. Conversely, extrinsic flaws can be introduced into dental ceramics by dental clinicians or technicians during grinding or polishing ceramic restorations [31], as well as because of masticatory force or parafunctional habits such as bruxism and clenching [32]. Such extrinsic damages may cause reduced strength, resulting in premature failure of dental ceramics [33]. Although dental 3Y-TZPs are strong enough to withstand the extrinsic damages [34,35], the damage coupled with LTD may become a strength limiting flaw. Nevertheless, there has been no systematic attempt to evaluate the influence of extrinsic stimuli on the strength of monolithic zirconia restorations encountered by LTD. To simulate the extrinsic flaws, Vickers indentation was applied to the 3Y-TZP that was aged in ambient atmosphere prior to the indentation. Although the indentation loads used in this study are slightly higher than the mastication load, a comparable damage may occur in procedure of grinding or polishing by dental clinicians or technicians during dental practices [33]. In this study, the extent of LTD was evaluated using the Rietveld refinement and the influence of preaging temperature on the strength of 3Y-TZP, aged in air and then indented, was investigated to elucidate the aging dependence of strength.

## 2. Materials and Methods

3Y-TZP powder was used for the specimens and the material information is listed in Table 1. The powder was pressed uniaxially at 125 MPa in a steel die, then pressed isostatically at 186 MPa to produce pellet-shaped green compacts (diameter: 20 mm, height: 1.5 mm). The pressed specimens were debinded for 55 h up to 985 °C, and then sintered for 2–20 h at 1550 °C on an alumina crucible in air. To prevent potential interaction between the specimens and the crucible, the top surface of the sintered specimens was used exclusively for XRD and indentation. After sintering, the diameter and thickness of the specimens were 15.7 ± 0.05 mm and 1.22 ± 0.04 mm, respectively. No surface treatment was performed prior to the experiments.

Initially, 20 specimens of each were sintered at 1550 °C for 2, 8, 14, and 20 h, and half of each group was indented at 9.8 N. After the initial trials, 350 pellets were fabricated by sintering at 1550 °C for 20 h and randomly divided into five groups (*n* = 70) based on aging temperatures of 130, 160, 190, and 220 °C, including as-sintered specimens for the control. Each group of specimens was subdivided again by 10 specimens for indentations at loads of 0, 9.8, 49, 98, 196, 294, and 490 N. All specimens were aged for 10 h at each temperature in air prior to the indentations. The microstructures of the sintered specimens, thermally etched for 15 min at 1500 °C, were observed using a scanning electron microscope (SEM, S4700, Hitachi, Tokyo, Japan) at ×30,000 magnifications with an accelerating voltage of 20 kV.

The bulk density of the fully sintered blocks was determined in accordance with ISO/DIS 18754 [36], using distilled water as the immersion liquid. The m-ZrO_2_ fraction was determined using Rietveld refinements from the XRD data for the specimens, which were collected on a Bragg–Brentano diffractometer (PANalytical, Almelo, The Netherlands) with a CuKα X-ray tube, a Bragg–BrentanoHD optical module, a position-sensitive PIXcel3D detector, and Soller slits (0.02 rad). The scan range covered 2θ = 20–90° at a step size of Δ2θ = 0.0217°. The data reduction and structure determination from the XRD data were performed using the HighScore Plus suite.

For the Vickers indentations, predetermined loads ranging from 9.8 to 490 N were indented for 10 s at the center of each specimen using a digital Vickers hardness tester (Highwood HWDV-7, TTS Unlimited Inc., Osaka, Japan). Immediately after unloading, a drop of silicon oil was applied on the indented surface to prevent possible humidity assisted-crack growth. The biaxial flexural strength was determined in accordance with the ISO 6872:2015 standard for dental ceramics [37]. The indented surface of each specimen was positioned to face the three supporting steel balls (diameter: 2.7 mm), positioned 120° apart on a support circle, then loaded at a crosshead speed of 1.0 mm/min using a universal testing machine (Instron 3365, Canton, MA, USA). To avoid contact damage, an ethylene–vinyl acetate foil was inserted between a flat punch (diameter: 1.4 mm) and specimen to ascertain an even contact.

Statistical analyses were performed using R software (version 3.6.2, R Foundation for Statistical Computing, Vienna, Austria). As the data showed non-normal distribution, they were analyzed using non-parametric statistical test, aligned rank transform analysis of variance (ART ANOVA), and multiple comparison using Bonfferoni post hoc test. The effect of independent variables such as the aging temperature and indentation load on biaxial strength, and the interaction of the two independent variables were determined. For all statistical analyses, a significance level of 5% was used.

## 3. Results

**Figure 1:**
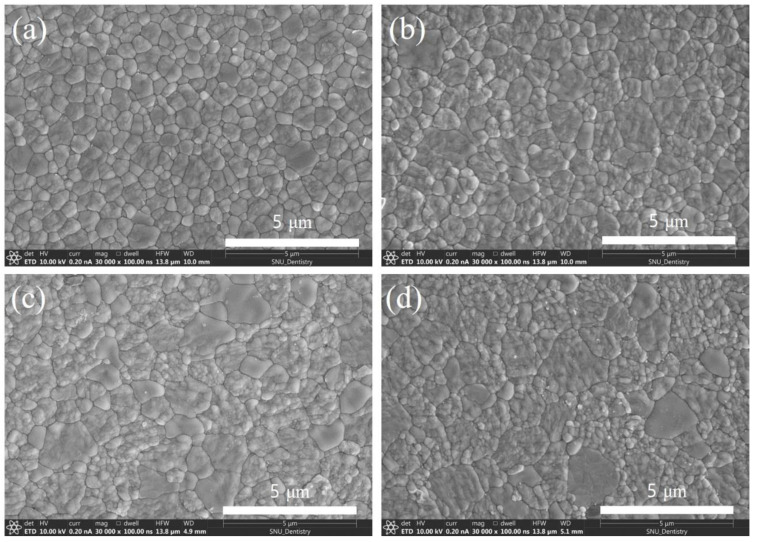
Scanning electron micrographs of 3Y-TZP sintered at 1550 °C for (**a**) 2 h, (**b**) 8 h, (**c**) 14 h, and (**d**) 20 h. The specimen, sintered for 2 h, shows fine grain size and well-defined grain boundaries. As the sintering time increases, the grain size increases and the grain boundaries became obscured by the disintegration of grains into pristine ones.

**Figure 2:**
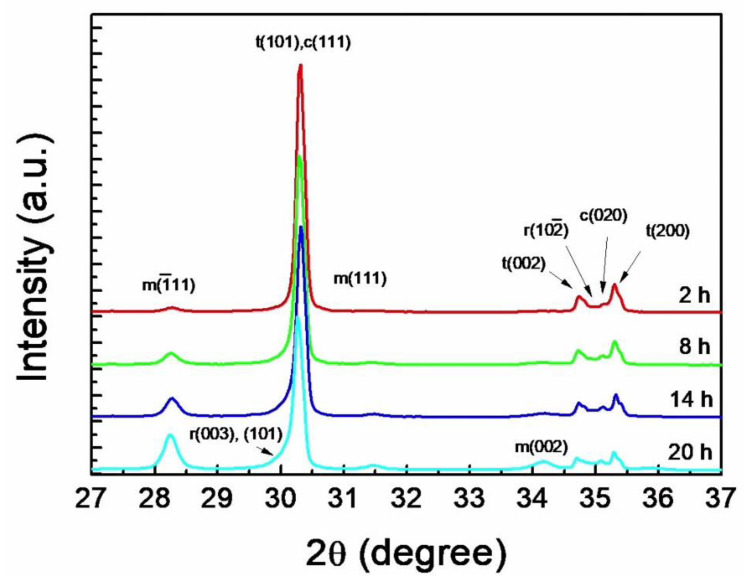
X-ray diffraction patterns of 3Y-TZP sintered for 2–20 h at 1550 °C. The (101) peak of t-ZrO_2_ is overlapped with (111) peak of c-ZrO_2_ and (003) and (101) peaks of r-ZrO_2_ to display an asymmetric broadening. The depth of broadening is proportional to the extent of t→m phase transformation.

The SEM micrographs of 3Y-TZP sintered at 1550 °C for 2–20 h are shown in Figure 1. After sintering for 2 h, the grain boundaries were well defined, and the size was determined to be 0.53 μm (Figure 1a) and increased to 0.72 μm after sintering for 8 h (Figure 1b). As time further increased, the grain boundaries became obscure because of pulverization of the grains into pristine grains. The unclear boundaries and pulverization made it difficult to determine the grain sizes after sintering longer than 14 h (Figure 1c,d). The XRD patterns of the specimens revealed that the t→m phase transformation extended with the sintering time as demonstrated by the increase in the peak heights for m-ZrO_2_ and the decrease in the peak intensity of t-ZrO_2_ in Figure 2. This suggests that the uneven microstructures were mainly due to the volume expansion accompanied by the t→m phase transformation and boosted because of thermal etching after polishing for SEM observations, which may ease the restriction that retained the pristine grains inside the grain boundaries.

In Figure 2, the increase in the phase transformation is manifested by the increased peak intensities of m-ZrO_2_ along with an asymmetric broadening of the (101) peak of t-ZrO_2_ with increasing sintering time. After sintering for 14 h the broadening of the peak at 30° becomes visible. The peak for c(111) is imbedded in the peak of t(101) and the peak for c(020) is shown as a separate peak at 35.2° between two t-ZrO_2_ peaks. The markers for r-ZrO_2_ related to the broadening are supposed to appear at 2θ of ~30° and ~35° but are not apparent because the extent of c→r phase transformation is limited. The peak intensity of m(111) at 31.5° for m-ZrO_2_ is so weak that the peak intensity ratio of m($\bar{1}$11)/m(111) is anomalously high compared to the ratio of 1.47 that is found in the ICDD # 00-037-1484 for m-ZrO_2_. In the XRD pattern for 20 h it is interesting to note that the peak intensity of m(002) at 34.2° is higher than that of m(111) even though the intensities are listed as 21 and 68, respectively. No peak overlapping for m-ZrO_2_ was observed in the entire scan range.

**Figure 3:**
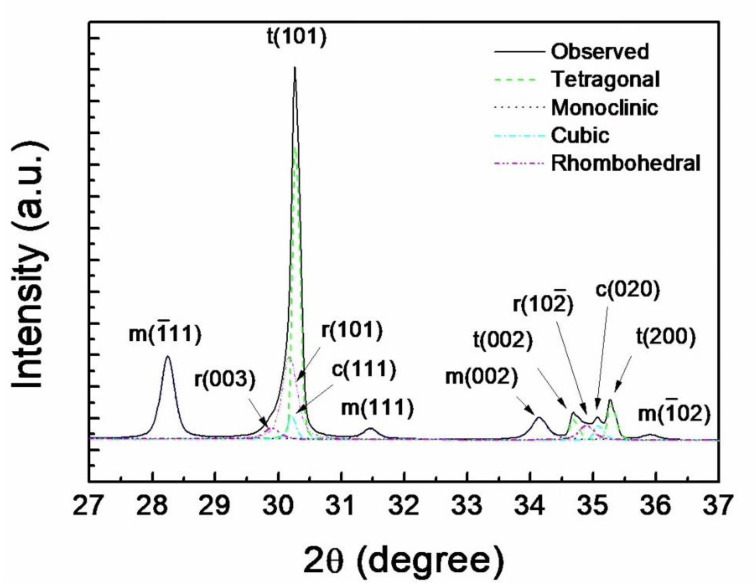
Deconvolution of asymmetric (101) peak of t-ZrO_2_ into (003) and (101) peaks of r-ZrO_2_ and (111) peak of c-ZrO_2_ from 3Y-TZP sintered for 20 h at 1550 °C. The overlapping becomes more evident at 2θ of ~35° where r(10$\bar{2}$) and c(020) are separated from t(002) and t(200). No overlapping for m-ZrO_2_ is observed.

In Figure 3 the t(101) peak at 30.2° from the 3Y-TZP sintered for 20 h at 1550 °C was deconvoluted into t-, r-, and c-ZrO_2_ phases by the Rietveld refinement. All peaks including the asymmetric peak were well identified with the given structure models including r- and c-ZrO_2_ phases. The Rietveld refinement revealed that the asymmetric peak is composed of r(003) at 29.8° and r(101) at 30.0° of r-ZrO_2_ and c(111) at 30.2° of c-ZrO_2_ besides t(101) of t-ZrO_2_. Thus, c-ZrO_2_ completely overlaps t-ZrO_2_ in the asymmetric peak. The deconvolution of the compound peak at ~35° showed the r(10$\bar{2}$) peak of r-ZrO_2_ at 34.8° embedded near the c(020) peak of c-ZrO_2_ at 35.1°. In this study the extent of t→m transformation was determined by the volume fraction ratio of m-ZrO_2_/(m-ZrO_2_ + t-ZrO_2_) based on the analysis by the Rietveld refinement procedure. The m-ZrO_2_ volume fractions in the specimens sintered at 1550 °C for 2, 8, 14, and 20 h (Figure 2) were calculated to be 15.4, 27.7, 39.6, and 44.6%, respectively.

**Figure 4:**
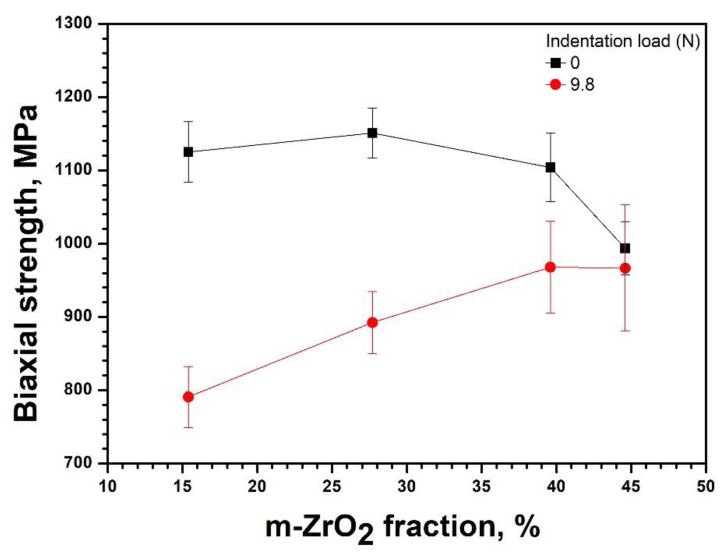
Variation of the biaxial strength of the as-sintered 3Y-TZP and the 3Y-TZP indented at 9.8 N as a function of m-ZrO_2_ fraction that is determined by sintering time. The biaxial strength of as-sintered specimens tends to decrease with m-ZrO_2_ fraction, but the strength of the identical specimens indented at 9.8 N increases with the fraction. The m-ZrO_2_ fractions were obtained by Rietveld refinement of 3Y-TZP sintered at 1550 °C for 2, 8, 14, and 20 h as shown in Figure 2.

The influence of the m-ZrO_2_ fraction, originated from the different sintering time (Figure 1), on the biaxial strength is depicted in Figure 4. The strength of the as-sintered specimens tended to decrease steadily upon the fraction. Conversely, the strength increased asymptotically with the fraction when 9.8 N of indentation was applied to the specimens. Although the strength of the indented specimen sintered for 2 h was lower than that of the as-sintered specimen by 334 MPa, the difference became nullified for the specimen sintered for 20 h. The decrease and increase in strength of the as-sintered and indented specimens, respectively, with the sintering time are likely influenced by both the enlargement of the grain size (Figure 1) and the increase in the extent of t→m phase transformation (Figure 2).

**Figure 5:**
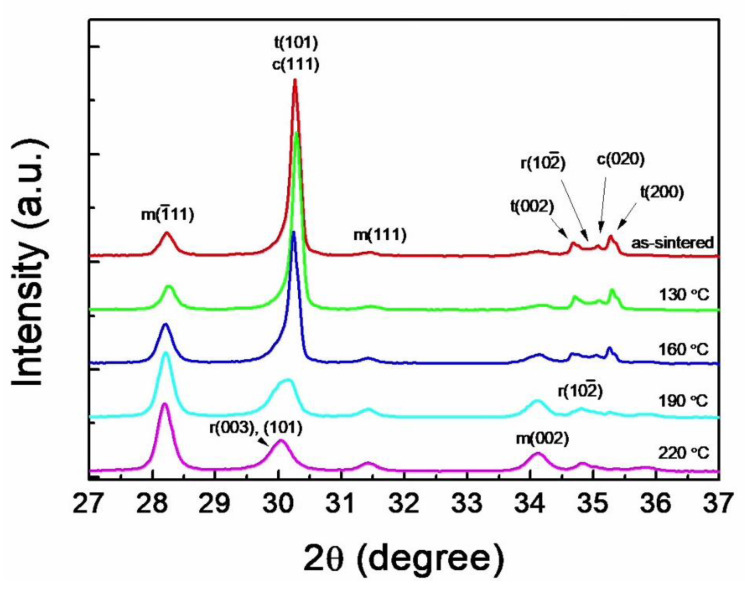
X-ray diffraction patterns of 3Y-TZP aged for 10 h at temperatures ranging from 130 to 220 °C after sintering at 1550 °C for 20 h. The pattern for the as-sintered specimen was added for comparison. The asymmetric broadening, associated with r-ZrO_2_ and c-ZrO_2_, increases with aging temperature and corresponds to r-ZrO_2_ at 220 °C suggesting that aging results in both t→m and c→r phase transformations.

The influence of aging temperature on the phase change was shown in Figure 5. The asymmetric broadening of the (101) peak of t-ZrO_2_ was also observed and increased in proportion to LTD. After aging at 190 °C the peak shape of t(101) disrupted to result in the transformation of 77.7 vol.% of t-ZrO_2_ into m-ZrO_2_. Concurrently, the r(10$\bar{2}$) became prominent at 34.8° and a marked preference of the m(002) orientation at 34.2° was observed. After aging at 220 °C the asymmetricity disappeared and 96.8 vol.% t-ZrO_2_ was transformed to m-ZrO_2_. The shift of the peak at 30.2° toward a lower 2θ and the disappearance of c(020) at 35.1° indicate that the t→m and c→r phase transformations were nearly completed. The m-ZrO_2_ volume fractions in the as-sintered and the specimens aged at 130, 160, 190, and 220 °C for 10 h were 35.6, 40.9, 52.2, 77.7, and 96.8%, respectively.

**Figure 6:**
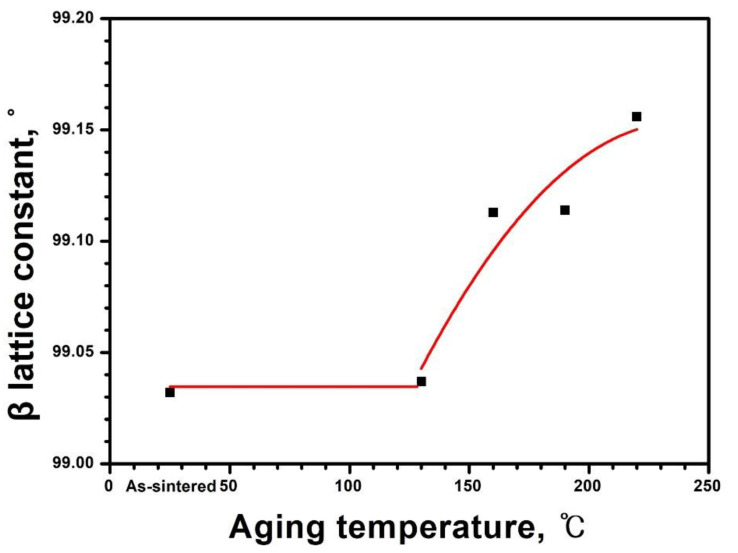
Variation of the β lattice constant of m-ZrO_2_ of 3Y-TZP sintered at 1550 °C for 20 h as a function of the aging temperature. The abrupt increase of β after aging at 160 °C indicates that the compressive residual stress in m-ZrO_2_ is reduced to some extent.

The Rietveld refinement revealed no significant changes in the lattice parameters of m-ZrO_2_ as the aging temperatures increased. However, the lattice constant of β, the angle between *a* and *c* axes, varied with LTD as depicted in Figure 6. The β of 99.03° for the as-sintered 3Y-TZP remained nearly unchanged up to 130 °C, then expanded upon further increasing the temperature suggesting that LTD is closely related to the variation of β. The lattice constants of the m-ZrO_2_ were calculated by the Rietveld refinement through all possible Bragg reflections in the corresponding crystal structures in the XRD pattern. Since it underwent a process of defining the correct peak position through correction of the zero-shift error and/or sample displacement error, the estimated standard deviation involved in β ranges from 0.0015 to 0.0036°.

The bulk density of 3Y-TZP sintered at 1550 °C for 20 h was 6.01 g/cm^3^, corresponding to 98.5% of the theoretical density (6.10 g/cm^3^) of 3Y-TZP. The density decreased slightly as the aging temperature increased from 130 to 220 °C in Table 2, where all specimens were aged for 10 h at each temperature. The decrease in density was concurrent with the increase in the extent of t→m phase transformation (Figure 5) because the theoretical density of m-ZrO_2_ (5.83 g/cm^3^) is lower than that of t-ZrO_2_.

**Figure 7:**
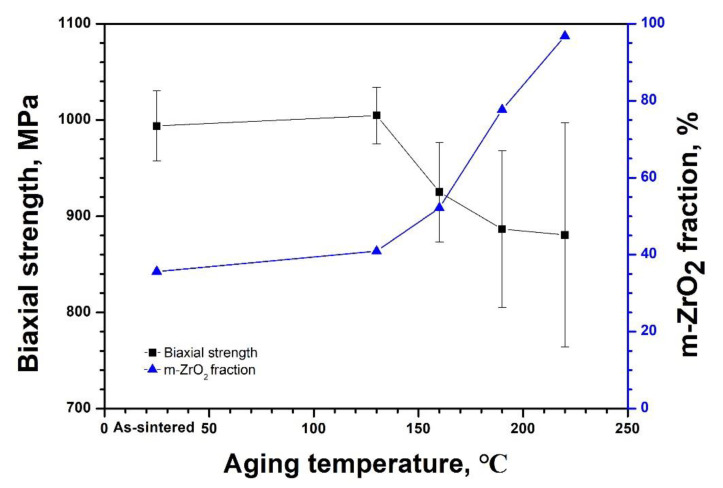
Biaxial strength and fraction of m-ZrO_2_ transformed from t-ZrO_2_ determined after aging for 10 h at temperatures ranging from 130 °C to 220 °C. The results demonstrate the degradation of strength with LTD.

In Figure 7 the fractions of t→m transformation for the specimens in Figure 5 were plotted along with the corresponding biaxial strength as a function of the aging temperature. As the aging temperatures increased, the m-ZrO_2_ content increased and the biaxial strength decreased, except for aging at 130 °C, showing a slightly higher strength than the as-sintered specimen even though the m-ZrO_2_ fraction was extended moderately from 35.6% to 40.9%. As the aging temperature increased further, the strength decreased abruptly in the same manner as the increase in the extent of t→m transformation.

**Figure 8:**
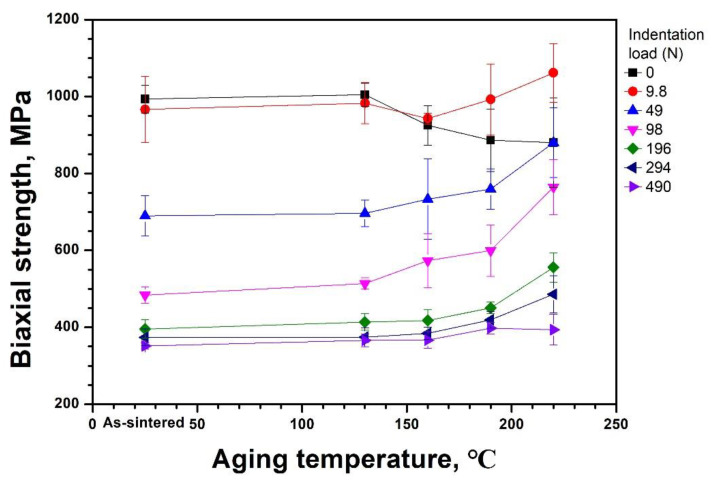
Variation of the biaxial strength of 3Y-TZP indented at loads ranging from 0 to 490 N as a function of aging temperature. The results show that the indented strength of preaged specimens increases with LTD. The specimens were aged for 10 h after sintering at 1550 °C for 20 h. The strength of as-sintered specimen is displayed at the scale of 25 °C.

In Figure 8 the involvement of the preaging temperature in the indented strength was demonstrated by the plot of the strength as a function of the temperature. The 3Y-TZP specimens were sintered at 1550 °C for 20 h, then aged for 10 h at 130–220 °C; the as-sintered specimen was included as a control. The specimens were indented at loads ranging from 9.8 to 490 N prior to the strength measurement and a set of specimens without indentation was added to facilitate comparison. The strength of the indented specimens tended to increase with the preaging temperature, and the increasing trend diminished as the indentation load increased. Similar results were also observed in Figure 4, where the biaxial strength of the specimens indented at 9.8 N increased with m-ZrO_2_ content, which was varied by sintering times at 1550 °C. Notably, the strength of the specimens, aged at 160–220 °C and then indented with a load of 9.8 N, was higher than the identical specimens without indentation. The contribution of indentation on the increase in strength became progressively prominent up to a load of 98 N, then became less noticeable upon further increasing the loads. Based on the statistical analysis, the aging temperature and indentation load significantly impacted the biaxial flexural strength 3Y-TZP (*p* < 0.05, Bonferroni post hoc test). Furthermore, there was significant interaction between two independent variables of preaging temperature and indentation load (*p* < 0.05, Bonferroni post hoc test), as listed in Table 3.

## 4. Discussion

This study was initially designed to examine the extent of the t→m phase transformation of 3Y-TZP for up to 100 h at a temperature range of 130–220 °C at 30 °C intervals in air. Primary tests showed that the LTD resistant 3Y-TZP containing 0.25 wt.% Al_2_O_3_, prepared by sintering at 1550 °C for 2 h, underwent an insignificant amount of phase transformation after aging for 100 h at the lowest intended temperature of 130 °C. Since it caused difficulty in determining the extent for aging times shorter than 100 h, the 3Y-TZP was sintered at 1550 °C for 20 h to expedite the transformation and was used for the remaining experiments. The 3Y-TZP, employed in this investigation, is known to be less vulnerable to LTD because the Al_2_O_3_ content of 0.25 wt.% decreases the grain boundary energy of t-ZrO_2_ to suppress LTD as a result of the grain boundary segregation of Al^3+^ ions [38]. Furthermore, this aging study was performed in ambient atmosphere to minimize the involvement of water species, which has been reported as the cause of LTD in numerous reports on this subject. This study demonstrates that LTD tolerant 3Y-TZP encounters t→m phase transformation in an ambient atmosphere, where no intentional water species are involved like autoclave treatments, as 3Y-TZP is stressed internally by the enlargement of grain size.

The t→m transformation governing LTD proceeds in air as long as the grain size is sufficiently large to cause sufficient residual tensile stress at the grain boundaries and corners [39]. The grain size dependence of the transformation in Figure 1 and Figure 2 stems from the fact that the local tensile stress concentration, due to the thermal expansion anisotropy (TEA) of the *a* and *c* axes of tetragonal zirconia lattice, scales with the grain size [39,40]. The dependency is manifested by the increased t→m phase transformation temperature and fracture toughness with the increase in grain size. The TEA in Y-TZP decreases with increasing Y_2_O_3_ content in ZrO_2_ [41] leading to the phase stability against LTD and consequently to a low fracture toughness of tetragonal zirconia. Conversely, TEA increases with the addition of pentavalent cation oxides, such as Nb_2_O_5_ and Ta_2_O_5_, to Y-TZP [42], resulting in phase instability favorable to LTD [7,8,43] and enhanced fracture toughness [8,44]. In this context, the stabilizer for t-ZrO_2_, which consequently prevents LTD, can be defined as oxides that reduce TEA when added to ZrO_2_ to form solid solutions. Due to the dominant contribution of residual stress to LTD, it occurs in environments where no water is involved, such as in vacuum and dried air [43] and moisture-free silicon oil [8], once the tetragonal solid solutions are stressed internally by high TEA, which is commonly accompanied by high *c/a* axial ratios of the tetragonal lattice [44]. Thus, in this study, the 3Y-TZP containing Al_2_O_3_, which prohibits LTD, experienced extensive LTD at ambient atmosphere because the residual stress was sufficiently large to trigger the phase transformation. These observations confirm that environments containing water or water vapor are not prerequisites for LTD but accelerate LTD [8,43].

Additionally, the residual stress influences the strength of 3Y-TZP in the t→m phase transformation, which can be envisaged from the strength of the specimens indented with 9.8 N in Figure 4. Without the indentation the strength decreased gradually with the increasing m-ZrO_2_ fraction due to the increase in sintering time. The decrease is likely resulted from a combined contribution of the increase in the grain size (Figure 1) in accordance with the Hall–Petch relationship and the t→m transformation (Figure 2). However, the increase in the indented strength with the grain sizes suggests that the main reason behind the decrease in the strength is the transformation related residual stress. An analogous trend was also observed when the specimen was aged at 130, 160, 190, and 220 °C for 10 h in Figure 7, where the influence of grain size is not involved. These observations consist with the reports that the strength decreases with LTD [25,26,27]. The decrease in the strength has been rationalized based on the argument that when the depth of the transformed layer is greater than the critical crack size of the specimen, determined by the linear fracture mechanics, the strength decreases in proportion to the m-ZrO_2_ fraction [45]. If this were the case, it would be hard to interpret the opposite trend that the strength of the identical but indented specimens increased with the fraction. This is because impressions by the indentation should behave as an additional defect besides the layer to aggravate the strength. Thus, an alternate rationale is required for the interpretation of the decrease in strength with the increase in the t→m phase transformation.

The possible involvement of the residual internal stress on the strength of the aged t-ZrO_2_ was further examined using Vickers indentation in Figure 8. After conducting Vickers indentation on the surface aged at various temperatures, the strength increased with the aging temperatures for all the specimens indented at loads ranging from 9.8 to 490 N. This result is analogues to the reports on the strengthening by LTD [22,23,24]. The increasing rate of strength with the aging temperature was highest at 98 N and reduced as the load increased. The strength of the specimens indented at 9.8 N was even higher than that of non-indented specimens at aging temperatures of 160–220 °C. The unexpected result was related to the residual stress around the indentation. The impression formed by the Vickers indent is generally described by a hemispherical compressively plasticized zone beneath the indent, surrounded by the elastic matrix. The elastic/plastic contact generates residual stresses around the indentation, which play a significant role in the fracture process of ceramics [46]. Particularly, the compressive residual stress originates from the constraint on the expanded indentation region from the unyielded material surrounding it [47]. Thus, the fracture toughness of indented brittle ceramics is given by the applied stress intensity factor and indentation residual stress intensity factor [48]. Herein, the influence of indentation on the strength of the aged 3Y-TZP is possibly related to the compressive residual stresses from both the indentation and the revelation of the compressive layer beneath the tensile surface.

The t-ZrO_2_ and m-ZrO_2_ grains on the surface of the aged specimens experience tensile and compressive stress, respectively [15,16]. The compressive residual stress can be manifested by the β lattice constant in Figure 6, which is significantly smaller than 99.22° of the constant of m-ZrO_2_ compiled in the ICDD # 00-037-1484. When an indenter is placed on the surface of the aged specimens, the residual compressive zone beneath the indenter and partially exposed compressive layer by the indentation contribute to retarded crack growth in the form of indentation residual stress intensity [48]. Furthermore, the m-ZrO_2_ on the aged surface may transform back to t-ZrO_2_ as the indentation expands and envelops the aged material in the highly compressive region beneath the indenter [47]. The decreased elastic modulus by LTD [49] may facilitate the envelopment because materials with low ratios of the modulus/strength easily accommodate the indentation volume into the plastically deformed zone beneath the indenter [50]. The freshly created compressive surface by indentation combined with the residual stress intensity factor may cause increased strength of the indented specimens in Figure 4 and Figure 8. The residual stress originates from hydrostatic compressive stress accompanying the t→m phase transformation in the plastic zone [51]. As the plastic zone size increases with the indent load, the residual tensile stress located on the edge of the impression, which is produced by mismatch tractions on the surrounding matrix by the plastically deformed zone [46], may play a role in the distraction of the compressive field. Consequently, the constructive influence of indentation on the strength of the aged specimens reduces at loads exceeding 196 N and nearly disappears at a load of 490 N. Additionally, the complete disappearance at a load of 490 N may be associated with the decrease in residual stress due to lateral cracking [48].

The stress-induced martensitic t→m phase transformation is characterized by an athermal and diffusionless process; however, the kinetics of LTD involve isothermal and diffusion-controlled phase transformation [8]. When the initial t→m transformation occurred, the shape strain comprising shear and dilational strains exerts opposing stresses in the surrounding matrix to place a local internal stress that could cause further stress-induced t→m transformation leading to the autocatalytic transformation [40]. This can occur immediately at the moment of the crack propagation to contribute to the phase transformation toughening. In LTD, however, the opposing stresses are accumulated by the isothermal t→m transformation through the diffusion of oxygen vacancies and may be accommodated to trigger the c→r transformation. This is supported by the fact that the c→r transformation is a stress induced phase transformation [52] and the c-ZrO_2_ content in 3Y-TZP decreases with prolonged aging time [20]. When the opposing stresses become large enough by aging at temperatures higher than 160 °C, the remnant shear stresses expand abruptly the β lattice constant in m-ZrO_2_ from 99.03 to 99.11° (Figure 6) to relieve the compressive stress in m-ZrO_2_ resulting in additional tensile residual stress on the aged surface. The sudden increase in β is likely related to the collapse of t(101) peak and the preferred orientation of m(002) as observed in Figure 5. This is analogous to the autocatalytic effect in a diffusion-controlled manner. As LTD proceeds, thus, the fractions of t-ZrO_2_ and c-ZrO_2_ decrease and those of m-ZrO_2_ and r-ZrO_2_ increase.

The m-ZrO_2_ fraction on the surface aged at 160 °C was 52.2% in Figure 7. The relaxation of the compressive stress is supported by the fact that the steady increase in the compressive stress in m-ZrO_2_ with aging was halted but the tensile stress in t-ZrO_2_ increased continuously when the m-ZrO_2_ fraction was higher than approximately 50% [15]. In this case the aged specimens lose their strength with an increase in LTD and the strength becomes subject to the influence of the indentation as shown in Figure 7 and Figure 8. Conversely, when the opposing stress is consumed only for the c→r transformation, the β lattice parameter remains nearly constant at about 99.03° and the aged strength increases with LTD possibly because the compressive stress in m-ZrO_2_ overwhelms the tensile stress in the untransformed t-ZrO_2_. This is the case where the indentation strength of specimens preaged at temperatures lower than 130 °C increases slightly with LTD in Figure 7 and Figure 8. The transition of LTD dependence of the strength was observed from 3Y-TZP subjected to accelerated aging at 134 °C, where the transition occurred after 14.7 h of aging [53]. It should be noted that the m-ZrO_2_ fraction that determines the dominancy of the residual stress state on the aged surface may not be fixed for 3Y-TZP but rather varies with the magnitude of the intrinsic or extrinsic residual stresses in specimens.

As previously mentioned, the influence of LTD on the flexural strength of Y-TZP is controversial [22,23,24,25,26,27,28,29,30]. Based on observations of the indentation dependence of the strength of aged 3Y-TZP, controversies surrounding the effect of aging on the strength of 3Y-TZP might be rationalized by considering the residual stress state on the surface of the aged specimens. The argument of residual stress can facilitate to envision the mechanism for LTD in such that the oxygen vacancies in t-ZrO_2_ under the residual tensile stress on the surface migrate into the bulk overcoming the activation barrier of ~83 kJ/mol along the residual stress gradient to relieve the internal stress in t-ZrO_2_ lattice [7,8,43]. This study demonstrates that the stress state can be envisaged by the measurements of the indentation strength of 3Y-TZP after aging. The surface residual stresses can be encountered, not only by the t→m transformation, but during the preparation of strength specimens such as the severity of grinding for dimensioning sample size, heat treatment after machining, abnormal thermal expansion mismatch between 3Y-TZP specimens and a ceramic substrate where the specimens are placed during sintering, and thickness of the bending specimens. In clinical situations, the occlusal adjustment of the restoration may be a cause of surface residual stress, which may influence the longevity of the restorations.

## 5. Conclusions

Even LTD resistant 3Y-TZP containing 0.25% Al_2_O_3_ underwent LTD in air, where no intentional water species were involved, once the internal residual stress is sufficiently large to induce t→m phase transformation. The transformation was accompanied by c→r transformation, which is responsible for the asymmetric broadening of the (101) peak of t-ZrO_2_ lattice. The β lattice constant of m-ZrO_2_ lattice was highly contracted by LTD demonstrating compressive residual stress state in m-ZrO_2_. The magnitude of β depended on the build-up of opposing stress in the surrounding matrix imposed by the t→m transformation. The influence of preaging temperature on the β value and the indented strength of 3Y-TZP suggested that as the m-ZrO_2_ is under a heavy compressive stress at relatively early stage of LTD, the aged strength increases with LTD, and as the compressive stress is relaxed after an extensive LTD, the strength deteriorates with LTD. The transition occurred at the m-ZrO_2_ fraction of 52% as determined by the Rietveld refinements.

## Figures and Tables

**Table 1:** Material information for 3Y-TZP utilized in the present study *.

Brand Name	Composition	Lot No.	Manufacturer
TZ-3YSB-E	Y_2_O_3_ 5.2 ± 0.5 HfO_2_ ≤ 5.0 Al_2_O_3_ ≤ 0.1–0.4 SiO_2_ ≤ 0.02 Fe_2_O_3_ ≤ 0.01 Na_2_O ≤ 0.06	S309336B	Tosoh, Tokyo, Japan

* as disclosed by the manufacturer.

**Table 2:** The bulk density of 3Y-TZP specimens aged for 10 h in air after sintering for 20 h at 1550 °C.

	Aging Temperature, °C				
	As-sintered	130	160	190	220
Bulk density, g/cm^3^	6.01 ± 0.02	6.01 ± 0.01	5.97 ± 0.02	5.97 ± 0.02	5.96 ± 0.01

**Table 3:** Summary of ART ANOVA for the biaxial flexural strength of indented 3Y-TZP after aging.

Source	*Df*	F Value	*p* Value
Aging temperature	4	42.194	<0.001
Indentation load	6	824.465	<0.001
Aging temperature × Indentation load	24	10.605	<0.001

## Data Availability

The data presented in this study are available on request from the corresponding author.
